# Infectious Causation of Abnormal Host Behavior: *Toxoplasma gondii* and Its Potential Association With Dopey Fox Syndrome

**DOI:** 10.3389/fpsyt.2020.513536

**Published:** 2020-09-16

**Authors:** Gregory Milne, Chelsea Fujimoto, Theodor Bean, Harry J. Peters, Martin Hemmington, Charly Taylor, Robert C. Fowkes, Henny M. Martineau, Clare M. Hamilton, Martin Walker, Judy A. Mitchell, Elsa Léger, Simon L. Priestnall, Joanne P. Webster

**Affiliations:** ^1^ Department of Pathobiology and Population Sciences, Royal Veterinary College, University of London, Hatfield, United Kingdom; ^2^ London Centre for Neglected Tropical Disease Research, Imperial College London Faculty of Medicine, London, United Kingdom; ^3^ National Fox Welfare Society, Rushden, United Kingdom; ^4^ Parasitology Division, Moredun Research Institute, Edinburgh, United Kingdom

**Keywords:** neurotropic, inflammation, fox, host, schizophrenia, *Toxoplasma gondii*, behavior, Dopey Fox Syndrome

## Abstract

The apicomplexan parasite *Toxoplasma gondii*, the causative agent of toxoplasmosis, can infect all warm-blooded animals. *T. gondii* can subtly alter host behaviors—either through manipulation to enhance transmission to the feline definitive host or as a side-effect, or “constraint,” of infection. In humans, *T. gondii* infection, either alone or in association with other co-infecting neurotropic agents, has been reliably associated with both subtle behavioral changes and, in some cases, severe neuropsychiatric disorders, including schizophrenia. Research on the potential impact of *T. gondii* on the behavior of other long-lived naturally infected hosts is lacking. Recent studies reported a large number of wild red foxes exhibiting a range of aberrant behavioral traits, subsequently classified as Dopey Fox Syndrome (DFS). Here we assessed the potential association between *T. gondii* and/or other neurotropic agents with DFS. Live, captive foxes within welfare centers were serologically tested for *T. gondii* and, if they died naturally, PCR-tested for vulpine circovirus (FoxCV). Post-mortem pseudo-control wild foxes, obtained from pest management companies, were PCR-tested for *T. gondii*, FoxCV, canine distemper virus (CDV), canine adenovirus type (CAV)-1 and CAV-2. We also assessed, using non-invasive assays, whether *T. gondii*–infected foxes showed subtle behavioral alterations as observed among infected rodent (and other) hosts, including altered activity, risk, and stress levels. All foxes tested negative for CAV, CDV, CHV, and DogCV. DFS was found to be associated with singular *T. gondii* infection (captives vs. pseudo-controls, 33.3% (3/9) vs. 6.8% (5/74)) and singular FoxCV infection (66.7% (6/9) vs. 11.1% (1/9)) and with *T. gondii*/FoxCV co-infection (33.3% (3/9) vs. 11.1% (1/9)). Overall, a higher proportion of captive foxes had signs of neuroinflammation compared to pseudo-controls (66.7% (4/6) vs. 11.1% (1/9)). Consistent with behavioral changes seen in infected rodents, *T. gondii*–infected foxes displayed increased attraction toward feline odor (n=6 foxes). These preliminary results suggest that wild foxes with DFS are infected with *T. gondii* and likely co-infected with FoxCV and/or another co-infecting neurotropic agent. Our findings using this novel system have important implications for our understanding of both the impact of parasites on mammalian host behavior in general and, potentially, of the infectious causation of certain neuropsychiatric disorders.

## Introduction

The ability of parasites to alter the behavior of their hosts fascinates both scientists and non-scientists alike. However, while there are now numerous examples of parasite-altered behavior among invertebrate hosts (1), there remain few clear examples of vertebrate host-parasite systems, despite their potential profound theoretical and applied implications. Research in this field has, however, begun to shift toward obtaining a mechanistic understanding of parasite-altered host behavior. Likewise, interest is gathering on the potential infectious causation of acute and chronic diseases, and in particular, the possibility that infection with neurotropic pathogen(s) may be a contributing factor in the etiology of some human psychiatric illnesses. *Toxoplasma gondii*, the causative agent of toxoplasmosis, is a highly successful apicomplexan protozoan parasite capable of infecting all warm-blooded animals. Between 20% and 80% of the global human population are thought to be seropositive (2). Cats and their wild relatives (*Felidae)* are the only known definitive hosts of *T. gondii*. Gametogenesis and sexual reproduction of parasites occurs within the small intestine of infected cats, resulting in the shedding of oocysts containing infectious sporozoites in the cat’s feces (3). Asexual reproduction in intermediate hosts (e.g., rodents and birds, likely to be predated by felines) and (accidental) secondary hosts (including humans, livestock, and other domesticated and wild mammals) is typified by rapidly dividing motile tachyzoites and slowly dividing sessile bradyzoites, the latter of which encyst in various tissues including the brain, heart, and skeletal muscle, potentially persisting for the lifetime of the host (4). In addition to congenital transmission (5), and potentially also sexual transmission (6), all hosts are primarily infected upon ingesting either oocysts (through contaminated food, soil or water), or bradyzoite-containing tissue cysts (through eating raw or undercooked infected meat, including *via* scavenging or predation). Since sexual reproduction of *T. gondii* can be accomplished only in felines, there are likely to be strong selective pressures on the parasite to evolve mechanisms to enhance transmission from the intermediate host to the definitive feline host. The predilection of *T. gondii* for the brain also places this parasite in a privileged position to specifically manipulate intermediate host behavior in order to enhance transmission (7, 8). On the same premise, the localization of *T. gondii* in the brain may be predicted to alter the behavior of other infected secondary host species. In this case, behavioral alterations do not occur *via* selective manipulation to enhance transmission but do so as a “by-product” of infection, even if of no current adaptive value to the parasite. Therefore, from an evolutionary selection perspective, the latter can thus be referred to as “parasite constraint” rather than “parasite manipulation” (9).

The severe sequalae of congenital infection, as well as post-natal or adult-acquired infections among immunosuppressed humans and animals, are well established (10, 11). However, there is now also a compelling and convincing body of evidence demonstrating, often subtle, changes in behavior associated with latent *T. gondii* postnatally and/or perinatally acquired infections across both rodent intermediate hosts, consistent with parasite manipulation (9) and human secondary hosts, consistent with parasite constraint (12). For example, both wild and laboratory-bred rats, *Rattus norvegicus*, infected with *T. gondii* exhibit higher activity or exploratory levels and, under particular conditions, an increased propensity to be trapped in cages relative to their uninfected counterparts (13, 14). *T. gondii*–infected rodents have also been shown to be less vigilant of predators (7, 8, 13–20) and, most notably, *T. gondii* infection appears to manipulate the perception of infected rodents to cat urine, whereby their strong innate aversion becomes a suicidal “fatal feline attraction” (21, 22). Such attraction appears to be specific to cat odor (urine), since infection has been demonstrated not to affect behavioral responses to odors of other non-predatory (such as rabbits) nor predatory (such as dogs or mink) mammals (23, 24). Similarly in humans with adult-acquired latent toxoplasmosis, research has demonstrated behavioral changes akin to that seen in infected rodent intermediate hosts (e.g. increased activity and decreased reaction times), and changes in human-specific traits including personality profiles (7, 12, 25–28). Furthermore, observational studies suggest that individuals with latent toxoplasmosis may be at increased risk of road traffic accidents compared to the uninfected general population, potentially consistent with increased risk-taking and/or diminished psychomotor performance (25, 29–32). *T. gondii*–infected male undergraduate students have even been demonstrated to rate the odor of domestic cats as more pleasant compared to uninfected individuals, suggesting that increased feline attraction may occur in infected secondary as well as intermediate hosts (33).

While the potential mechanism(s) of action regarding *T. gondii’*s ability to alter host behavior remains largely unknown, as is the situation across parasite-induced behavioral change in vertebrates in general (34), evidence from rodent models suggests that this behavioral alteration is specific to *T. gondii* rather than resulting from a generalized response to parasitic infection. For example, no such behavioral changes were observed in rodents infected with other directly transmitted parasites infections, including those associated with encephalitis, and all other key indicators of fitness, from body condition to social status, have been demonstrated to be indistinguishable from those of uninfected rodent (19, 35). There is, however, gathering evidence on the role of neuromodulation in behavioral change associated with *T. gondii* infection across both rodents and humans (36). Recent *in vitro* and *in vivo* research shows that, for example, *T. gondii* alters dopamine levels within the host brain. Moreover, it has been shown that the parasite’s genome contains two genes which encode tyrosine hydrolase, the enzyme which converts tryptophan to the dopamine precursor, L-DOPA (34, 37, 38). Expression of one of these genes is induced in the bradyzoite stage, resulting in a concentration of dopamine up to 3.5 times higher in *T. gondii*–infected compared to uninfected cells (38). *T. gondii*–infected cells often occupy key positions at functional synapses (39) and may therefore have considerable local influence on dopaminergic signaling. These findings are therefore consistent with a higher prevalence of schizophrenia in *T. gondii* latently infected populations (40–42), since dopamine dysregulation is a key characteristic of schizophrenia (43).

Physiological changes resulting from infection in intermediate rodent hosts likely have broader implications for infection of other host species. While rodents may be a suitable short-lived intermediate host model for understanding *T. gondii*-host interactions—and specific manipulation—one may predict similar behavior changes but also a range of other nonadaptive “unintended” consequences in longer lived hosts. Accordingly, analogous to the aberrant behavior of infected rodents, behavioral changes have been documented in, for example, California sea otters, *Enhydra lutris nereis*, where those with moderate to severe toxoplasmic encephalitis are at a 3.7 times higher risk of shark attack than *T. gondii*-uninfected conspecifics (44). Similarly, in humans, mounting evidence has associated *T. gondii* infection with a range of neurological disorders including addiction (42), bipolar disorder (42, 45), epilepsy (46–48) and most compellingly, schizophrenia (9, 40–42, 49–51). Indeed, the association of *T. gondii* with schizophrenia is of substantial current concern (21, 41, 42, 50) and one that cannot be thoroughly investigated given current animal models. While rodent studies to date, from both the laboratory and field, have provided a great deal of information relevant to the *T. gondii* model of schizophrenia [e.g. (9, 50)], there remains a need for further work on infections in naturally longer-lived mammalian hosts (relative to rodents), especially those likely to be repeatedly exposed to *T. gondii* from multiple sources.

The red fox *Vulpes vulpes* may provide key research opportunities to further understand the impact of *T. gondii* (and co-infecting neurotropic pathogens) on the behavior of longer-lived hosts for several reasons. Wild foxes often have high seroprevalences of *T. gondii* with for example, 35% seroprevalence found in populations in Austria (52) and 100% in Czech Republic (53). This is consistent with high levels of exposure to the parasite often seen in human populations: for example in Brazil, human population seroprevalence can reach up to 80–90% (54, 55), in France 89% (56) and in Northern Ireland 85% (57). Wild foxes also have longer lifespans relative to the commonly studied rodent animal models, allowing time for the accumulation of by-product effects of neurotropic infection that are seen in humans. Similarly, akin to human transmission, foxes acquire “trickle” infections—low level persistent re-exposure to the parasite—from a variety of sources, including *via* accidental ingestion of oocysts (e.g. *via* environmental contamination of food, water sources) and bradyzoites (via carnivory of a variety of infected wildlife). Perhaps, however, most notable of all, a presentation of clinical neurological signs has been recently characterized in red foxes, coined Dopey Fox Syndrome (DFS), for which the causative agent(s) remain(s) undetermined (58). Wild foxes with DFS have been reported to exhibit a range of abnormal behavioral traits, many of which are consistent with behaviors seen in *T. gondii*–infected rodents, including an apparent lack of fear and increased affection (59). Further pathological behavioral symptoms observed within DFS foxes include, but are not exclusive to, constant pacing, facial muscle twitching and anorexia. A minority of foxes with DFS also have encephalitis as well as visual abnormalities and/or blindness (59), consistent with ocular *T. gondii* infection (60).

Alternative, though not necessarily conflicting, hypotheses are that DFS may result from infection with an alternative infectious agent or from co-infection of *T. gondii* with another neurotropic agent in a “two-hit” manner (50). A two-hit model, which could encompass either gene-environment interactions and/or the combined effects of two co-infecting agents, has been proposed for certain *T. gondii*-associated behavioral alterations in animals and for some human neuropsychiatric disorders (50). For example, in a large cohort study, co-infection with *T. gondii* and other neurotropic agents, including CMV, measles, and vaccina viruses, but not singular infections, predicted an 18% to 34% increased risk of schizophrenia (61) [for meta-analyses see (62, 63)]. Several infectious inflammatory encephalitides of canids have been reported, including, for example, rabies virus, canine distemper virus (CDV) and a recently detected Borna disease virus (BoDV-1) (64–66). Members of the families *Adenoviridae* and *Herpesviridae* are also known to cause neurologic signs as part of a systemic disease process (67). Notably, circovirus is a pathogen that has been recently reported to neurologically affect foxes, with a recent study demonstrating a high prevalence of circovirus infection among UK wild foxes with unexplained meningoencephalitis (59). This fox-specific circovirus (FoxCV) is distinct from the dog circovirus (DogCV) that infects the domestic dog (*Canis familiaris*) (68). As circoviruses are known to have immunosuppressive effects (69), one could predict that these infections may occur in concert with other neurotropic agents and may therefore be associated with behavioral alterations in hosts.

Red foxes may therefore be a highly appropriate natural system to help elucidate the potential impact of *T. gondii* on longer-lived host organisms (such as humans), as well as to increase our understanding of the impact of neurotropic pathogens on mammalian host behavior in general. Here, we present preliminary data from a series of recent pilot studies, which aimed to test the hypotheses that DFS in wild red foxes is caused by: (a) *T. gondii* infection; (b) another neurotropic agent;and/or (c) *T. gondii* co-infection with another neurotropic agent (part of the “two-hit” hypothesis). We addressed these questions using a battery of diagnostic tests and non-invasive behavioral assays, comparing results between (i) foxes with DFS and other foxes maintained in welfare sanctuaries; and (ii) “pseudo-control” foxes provided by local pest control agencies.

## Materials and Methods

### Ethical Approvals

Full ethical approvals were provided by the Royal Veterinary College Clinical Research and Ethical Review Board (CREB) (URN numbers: 2018 RP2_004-2 and 2017 RP2_1768-3). As all behavioral studies performed on captive foxes were non-invasive and non-stress inducing, and no animals were euthanized for the research, there was no need for home office PIL or PPL licensing. Captive foxes were those caught from the wild, brought into and—where rehabilitation and release were not possible—maintained within large outdoor enclosures within local fox sanctuaries. Serological material from living foxes was collected by local veterinarians as part of routine health screening. Additional material for identifying alternative infectious agents was supplied by routine fox cadavers, brought into the RVC from local pest control services and any foxes dying or being humanely euthanized independently from this research within the collaborating fox welfare sanctuaries (National Fox Welfare Society and the Gin Pat Trust).

### Fox Populations

During the study period, the rescue centers had fewer than 30 foxes in each center, both of which are located in Northamptonshire, UK. Foxes arrived at these sanctuaries when they have been found injured or diseased; many had been involved in road traffic accidents or had neurological abnormalities indicative of DFS. The pens were a variety of sizes but averaged approximately 5 × 10 m and housed between two to six foxes. Foxes showing signs of illness were serologically tested for *T. gondii* and other infections as part of their general health screening. Foxes that were returned to fitness were released *via* hard release sites at their point of capture, or *via* soft release sites by opening the door to their pen. Cadavers (n=9) were obtained opportunistically from captive fox populations following humane euthanasia only in cases where welfare was compromised and where recovery was deemed not possible by consulting veterinarians.

A London-based pest control company provided cadavers (n=74). We used these wild caught foxes as a proxy for a “healthy” control group for comparison to the captive foxes. Hereafter we will refer to this group as pseudo-controls, since we acknowledge that foxes shot by pest control may be, for example, younger and/or less risk-averse relative to the average free-living wild fox population.

### Necropsy Examinations

A total of 83 foxes underwent full gross necropsy examination (74 pseudo-controls and nine captive foxes). A full external examination, which included sexing and ageing (70) was performed on all foxes. Gross lesions and signs of parasitic infection seen during the necropsy examination were recorded. Tissues were sampled from the brain (71). The head was detached by breaking the atlanto-occipital joint. The brain was externalized, and samples were taken of rostral cerebrum, mid cerebrum including hippocampus and amygdala and cerebellum including brainstem. The samples were then divided to be either: i) frozen at −80°C (and/or placed in RNAlater) for subsequent PCR testing; or ii) fixed in 10% neutral-buffered formalin for subsequent pathology.

### Determining Fox Infection Status

#### Sample Preparation

DNA was extracted from approximately 1 to 2 g homogenized cerebrum of 83 fox brains (74 pseudo-control samples and nine captive samples) using a previously described protocol (72). Extraction controls, processed identically to homogenized tissue, were used to monitor for potential cross-sample contamination.

RNA extractions from brain tissue for detecting other potentially co-infecting neurotropic agents were performed using the RNeasy Mini Kit (Quiagen) according to the manufacturer’s instructions, with some modifications as follows. After determining unacceptable levels of contamination *via* spectrophotometry for 30 mg brain tissue in 600 μl RLT buffer (excluded from analyses), 200 μl of the tissue-in-saline homogenate prepared for DNA extraction was mixed with 600 μl RLT buffer. RNA was eluted with 50 μl nuclease-free water and stored at −80°C.

Reverse transcription was performed to a reaction volume of 20 μl. Final reaction concentrations were as follows: 1× ImProm-II reaction buffer (Promega), 3 mM magnesium chloride, 0.5 mM dNTPs, and 1 µ ImProm-II Reverse Transcriptase (Promega). Briefly, 5 μl of RNA was incubated with 1 μl (0.5 μg) random hexamer primers at 70°C for 10 min, and then cooled on ice for 5 min. Reactions were incubated with Master Mix at 37°C for 1 h. cDNA was stored at −20°C for short-term and −80°C for long-term storage.

#### *T. gondii* Serology

To test the hypothesis that the aberrant behavior of foxes with DFS is associated with *T. gondii* infection (either alone or in combination with other neurotropic agent(s)), the seroprevalence of *T. gondii* in 21 captive foxes was evaluated in relation to behavioral profiles. Sera were obtained from live captive foxes by local veterinarians/rescue centers undertaking routine health screening testing (and/or at necropsy if any fox required euthanasia). *T. gondii* antibodies (IgG and IgM) were detected using a combination of commercial enzyme-linked immunosorbent assays (ELISA) (IDEXX) and Indirect Latex Agglutination Tests (ILAT) (Toxoreagent; MAST). For both the ELISA and ILAT, titers of ≥1:32 were considered positive [full details, including validation, has been published previously (21)].

#### *T. gondii* PCR

A nested PCR was performed using DNA extracted from 83 fox brains (74 pseudo-control samples and nine captive samples), targeting the internal transcribed spacer 1 (ITS1) region between the 18S and 5.8S rRNA genes of *T. gondii*. PCR amplifications were carried out in quadruplicate to improve sensitivity, using conditions described previously (72) (**Tables 1**, **2**). Extraction controls as well as negative and positive controls were included in each PCR.

**Table 1:** Primer and probe sequences for PCR assays.

Assay	F primer, R primer, probe	Sequence 5′ to 3′	Reference
Reference gene	GAPDH1 GAPDH2	GCC AAA AGG GTC ATC ATC TC GGC CAT CCA CAG TCT TCT	(73)
CAV-1, -2	VP1 VP2	CTG GGC GGG ATT TAG AGG GTG G CAA GGG CGT GGG CGG AGT TAG A	(74)
CDV	DISTF (p1) DISTR (p2)	ACA GGA TTG CTG AGG ACC TAT CAA GAT AAC CAT GTA CGG TGC	(74, 75)
CHV	HERP1 (CHV-1) HERP2 (CHV-2)	AAG AGC TCG TGT TAG TGA AAA T TAA ACC CGC TGG ATG ATA C	(74)
DogCV	Dog-CV-Forward Dog-CV-Reverse Dog-CV-probe	CCT GCG AGA GCT GCT CCT TAT AT CTC CAC TTC CGT CTT CCA GTT C FAM-TCC GGA GAT GAC CAC GCC CC- TAMRA*	(76)
FoxCV	VS756 VS757 VS758	TCC GAG ATA GCC GGC GTG GTA CCC GGC CAC AGA TCA AGT ACT TA FAM-ATC CAA CTC CGG AGG AGG AGG A-TAMRAł	(59)
*T. gondii* first round	NN1 NN2	TCA ACC TTT GAA TCC AAA CGA GCC AAG ACA TCC ATT	(72)
*T. gondii* second round	Tg-NP1 Tg-NP2	GTG ATA GTA TCG AAA GGT AT ACT CTC TCT CAA ATG TTC CT	(72)
*A. vasorum*	Nad3-F1 Nad3-R1	ATC GTG AGA TAG AAT TGT TTA TCT TG CCA ACT CTA CAC CAA TCA CAT CAA C	(77)

*5′ FAM reporter dye and 3′ TAMRA quench used for both qPCR assays.

**Table 2:** Target genes and cycling conditions for PCR assays.

Assay	Target gene	Amplicon size (bp)	Cycling parameters*	Reference
Reference gene	GAPDH	250	35 cycles: 95°C—1 min 55°C—40 s 72°C—1 min	(73)
CAV-1, -2	Capsid protein	704	35 cycles: 95°C—1 min 60°C— 40 s 72°C—1 min	(74)
CDV	Nucleoprotein	287	35 cycles: 95°C—1 min 55°C—40 s 72°C—1 min	(74, 75)
CHV	Homologous region to UL37 of HSV-1**	494	35 cycles: 95°C—1 min 49°C—40 s 72°C—1 min	(74)
DogCV	Rolling circle replicator initiator protein gene	66	Initiation: 94°C—2 min 40 cycles: 94°C—15 s 60°C—1 min	(76)
FoxCV	Rolling circle replicator initiator protein gene	126	Initiation: 94°C—2 min 40 cycles: 94°C—15 s 60°C—1 min	(59)
*T. gondii*	ITS1	227	35 cycles: 95°C—1 min 55°C—1 min 72°C—1 min	(72)
*A. vasorum*	Full NADH3 and partial rrnL genes	438	40 cycles: 95°C—30 s 55°C—30 s 72°C—1 min	(77)

*Unless otherwise indicated, each round began with a denaturation step by heating to 95°C for 5 min and ended with a final extension at 72°C for 5 min. Cycling conditions were similar for rounds 1 and 2.

**Herpes simplex virus type 1.

#### (Co)-Infection With Other Neurotropic Agents

To test the hypothesis that the aberrant behavior of foxes with DFS is associated with infection with a neurotropic agent other than *T. gondii*, either alone or in combination with *T. gondii* (two-hit model), we developed a multiplex assay which was performed on nucleic acid extracted from 18 fox brains (nine of each pseudo-controls and captives). The following pathogens were targeted due to their documented high prevalence in wild foxes and/or ability to cause neurological symptoms in other host species, using serological analyses and in-house PCR assays: canine-specific circovirus (DogCV) (76), the novel fox-specific circovirus (FoxCV) (59), canine herpes virus (CHV), canine distemper virus (CDV) (75) and infectious canine hepatitis caused by canine adenovirus type 1 and 2 (CAV-1, -2) (74). The reference gene glyceraldehyde-3-phosphate dehydrogenase (GAPDH) was amplified from all tissue samples to ensure the quality of nucleic acid extractions (73). A nested PCR was added to the protocol to detect *Angiostrongylus vasorum* after finding evidence of parasitic infection.

Where positive controls were not available for DogCV, and FoxCV PCRs, plasmids were constructed using the Invitrogen GeneArt service (ThermoFisher Scientific). Conventional PCR reactions were performed with 2 μl cDNA or gDNA to a final reaction volume of 50 μl, with the following reagent concentrations: 1× GoTaq reaction buffer (Promega), 0.5 μM each forward and reverse primers, 0.2 mM dNTPs, and 1 unit/μl GoTaq enzyme (Promega). Magnesium chloride concentrations varied as follows: GAPDH (2.5 mM), CAV (1.5 mM), CDV (2.5 mM), CHV (2 mM) and *A. vasorum* (1.5 mM) (77). Reagent concentrations for qPCR reactions were as follows: 1× JumpStart Taq ReadyMix (Sigma-Aldrich), 0.5 μM of each forward and reverse primers, 0.25 μM of the appropriate conventional hydrolysis probe, and 1 μl gDNA to a final reaction volume of 50 μl. Primer and probe specifications for all PCR reactions are listed in **Table 1**.

All conventional PCR assays were performed using GoTaq with an initial step of 95°C for 5 min and a final extension of 72°C for 10 min. All other cycling parameters, including those for qPCR assays, can be found in **Table 2**. End-point PCR products were visualized on agarose gel for all relevant targets (all but DogCV and FoxCV; **Table 2**). For qPCR targets—DogCV and FoxCV—positive control plasmids were validated, and a 10-fold dilution series was established to obtain an absolute detection limit and an internal standard curve for qPCR analysis. Brain tissue samples that amplified by 36 cycles (100–1000 template copies) were considered positive, with any signal obtained between 36 and 38 considered potentially positive.

#### Sequencing

FoxCV qPCR products from two individuals (N2 and N3) were sent to the Medical Research Council Protein Phosphorylation and Ubiquitylation Unit (MRC PPU) for sequencing. Two reactions were analyzed: one with each forward (VS756) and reverse (VS757) primers.

### Brain Histology

We next aimed to determine whether the aberrant behavior of foxes with DFS was associated with neurotropic inflammation, and whether this inflammation was associated with specific neurotropic agents. To do this, inflammation was assessed in relation to group (pseudo-control and captive) and species of infecting neurotropic agent. Fixed brains were sectioned transversely including representative portions of cerebral cortex, with the inclusion of midbrain with thalamus and hypothalamus, cerebellum and brain stem. Sections were cut at 4 µM (RVC Diagnostic Laboratories) and brain tissues stained with hematoxylin and eosin, Giemsa and Periodic acid–Schiff. Sections were systematically evaluated for: a) distribution, degree, and character of inflammation and degenerative change; and b) the presence of parasite cysts or free *T. gondii* zoites.

To compare brain inflammation between pseudo-control and captive foxes, brain sections were taken for histological analysis from *T. gondii* positive and negative individuals of the same age, sex, and body condition score, for a total of 15 samples (nine pseudo-controls and six captives). Two slides were prepared for each brain, one containing the amygdala and one containing the nucleus accumbens. The former brain region was included due to its known association with emotional responses; including, notably, the role of the amygdala in fear processing (37). Likewise, though inconsistent, there is evidence of a potential tropism of cysts for amygdalar regions, which may in turn be associated with host behavioral changes (23, 78). The nucleus accumbens was also included due to its role in motivation, reward, pleasure, and fear and its predicted—though inconsistently shown—involvement in *T. gondii*-altered behavior (78).

Slides from the nucleus accumbens were made following an incision rostral to the thalamus and caudal to the olfactory bulb. Amygdala slides were made following cutting through the center of the rostral quarter of the thalamus. Stained sections were taken for microscopic examination by a board-certified veterinary pathologist.

### Fox Behavior in Relation to *T. gondii* Infection Status

To determine whether *T. gondii*–infected captive foxes displayed behavioral alteration similar to those seen in *T. gondii*–infected intermediate rodent hosts, a modified, ethically appropriate form of the **“**fatal feline attraction” behavioral assay was performed within large outdoor enclosures (20–22). Testing for FoxCV could not be performed in live foxes since no specific serological test yet exists, and current DogCV serology does not cross-react to FoxCV (see Results). Of particularly pertinence is the observation that several of the *T. gondii*–infected foxes admitted to rescue centers had facial wounds indicative of cat attacks. Whether this increased proximity to cats may be simply related to, for instance, increased scavenging or increased risk behavior, or akin to the *T. gondii*-specific “fatal-feline-attraction,” was yet to be determined.

We recorded the continuous exploratory behavior of six randomly selected *T. gondii* seropositive captive foxes in 5^2^ m pens (or matched equivalent). Following our previously published protocol (22), the ground was covered with woodchips and the layer changed between each test iteration. Assays were initially performed between 10:30–12:30 and 14:30–16:30, however due to low fox activity levels and the crepuscular nature of the animals, assays were subsequently performed between 19:00 and 21:00.

The pens were split into four equal sized quadrants. Each quadrant contained a covered area for hiding, a water bowl and objects for behavioral enrichment (e.g. tunnel or stuffed toy). There was a neutral zone in the middle of the pen with no scent, which contained the drinking water, food, and most behavioral enrichment objects. The total time an individual spent in a zone was recorded (continuous sampling) and the zonal location of the individual at the end of each 5-min time interval (point sampling) was also recorded (79).

Dog and cat urine were obtained from veterinary practices. Dog urine was chosen because it has been shown that fatal feline attraction is specific to felines rather than being a general response to mammalian predators (20). Moreover, dog odor has also been used before as a control scent in previous “fatal feline attraction” studies in mammals (20, 23, 80). By using this approach, we also avoided including odor from a fox prey animal (e.g. rabbit). Species-specific urine was pooled to minimize inter-individual differences, since castrated cats have been shown to be less attractive to *T. gondii*–infected rats (81–83). The water bowls of two quadrants were mixed with 20 drops of dog urine and the water bowls of the remaining two quadrants mixed with 20 drops of cat urine. Bowls containing cat urine were placed diagonally opposite to one another to limit bias due to foxes avoiding zones near to the observer.

Foxes were observed for 2 to 4 h to assess baseline behavior. Then, over 2 h we recorded the relative amount of time each fox spent in different zones using a continuous sampling approach (79). Wherever possible, the observer was blinded to *T. gondii* infection status of individuals. Behaviors such as spinning, grooming or digging were noted. Control experiments were performed by observing the behavior of foxes in the absence of dog or cat urine in water bowls. Foxes were observed for a total of 42 h (18 h control group and 24 h treatment group).

### Biomarkers of Stress in Relation to Fox *T. gondii* Infections Status

While it has been proposed that *T. gondii* infection results in the alteration of anxiety levels, which may be responsible for behavioral changes, to our knowledge, no studies to date have explicitly tested this question. As chronic cortisol may be predicted to be an indicator of differential long-term anxiety profiles, we examined whether this phenomenon was observed in secondary fox hosts by detecting chronic stress levels through cortisol competitive immunoassays performed on fur samples (Salimetrics, #1-3002). Fur samples were chosen since it has been demonstrated that fur (and hair) reliably indicates long-term average cortisol levels, instead of providing a measure of acute stressors as do other samples including blood and saliva (84–86); the latter of which would be expected to be particularly biased since sampling methods for obtaining blood or saliva can stress the animal and lead to temporary cortisol release, independent of its longer term profile (87, 88).

A minimum of two 1^2^ inch samples of fur were taken from the thigh muscle region of each animal, at consistent distances from the hair follicle (to minimize sampling variation in cortisol concentration across the hair length) and stored at −20°C. The color of each fur sample was recorded, as this may impact the cortisol reading. Blood-stained regions of fur were avoided. Fur samples were ground using a Retsch MM300 ball mill (30 rotations/s, 40 min). The ground fur was added to 2 ml methanol and left overnight at room temperature for 19 h on a horizontal orbit microplate shaker (0.12” orbit, 150 ± 50 rpm). The methanol was placed in Eppendorf tubes and tube lids pierced to allow evaporation to dryness (50°C, 2 h). Samples were re-suspended in 100 μl assay diluent and loaded onto a plate coated with cortisol antibody and horseradish peroxidase conjugate. The rest of the assay was performed according to the manufacturer’s instructions (Salimetrics, #1-3002). To calculate the intra-assay coefficient of variance, an internal control cortisol standard was measured several times, giving a value of 9.03%.

### Statistical Analyses

All statistical analyses were performed in R (89). We used chi-square tests to assess the difference in the proportion of pseudo-control and captive foxes testing positive for: *T. gondii* infection alone, infection with alternative neurotropic agent(s), and *T. gondii* co-infection with another neurotropic agent. Chi-square tests were also used to compare the proportion of pseudo-control vs. captive foxes with evident signs of histological inflammation according to infection with specific neurotropic agents. We used the Friedman test to analyze the amount of time *T. gondii*-positive foxes spent in cat odor zones relative to dog odor zones in behavioral assays, in order to account for the non-normal data distribution and the repeated measures performed on individual. A chi-square test was performed to determine differences in cortisol concentration according to *T. gondii* infection status. For analyses using matched pairs of foxes, Wilcoxon signed-rank tests were used. Due to small sample sizes, test conditions for conventional chi-square tests could not be satisfied. Hence, to calculate p values Monte Carlo simulations were performed—each with 1000 replicates—by creating a reference distribution through random sample generation and comparison of our data to this reference (90). For this reason, degrees of freedom were not specified for this test.

## Results

### *Toxoplasma* *gondii* and Alternative (Co-) Infectious Agent Status

To test whether DFS was associated with *T. gondii* infection, we compared the prevalence of infection in captive to pseudo-control populations. Overall, 6.8% (5/74) of pseudo-control foxes and 33.3% (3/9) of captive foxes tested positive for *T. gondii* by PCR, suggesting quite strong evidence of a difference between groups (*χ* ^2^ = 6.51, p = 0.037). Furthermore, 66.7% (14/21) of captive foxes (longitudinally serologically tested for *T. gondii* infection) tested seropositive for *T. gondii* exposure by ELISA during at least one time point. Interestingly, two foxes showed evidence of IgG seroreversion; one of these foxes had two sequential tests performed over 10 days.

We next considered whether DFS may be instead associated with neurotropic agent(s) other than *T. gondii* (or in combination with, see below) in a small sample of pseudo-control and captive foxes, matched for age, sex, and body condition score, in addition to identifying histological signs of inflammation (**Table 3**). Only captive foxes with severely compromised welfare were humanely euthanized (independently of the current study), and hence, PCR testing could not be performed on living captives. Of the captive foxes (where N denotes neurologically affected (captive) foxes), N1, N5, and N9 were negative for all infectious agents tested, suggesting an alternative and thus far undetermined, possibly non-infectious, etiology for their clinical signs. Formalin-fixed samples were not available from N1 and N5 for histological analysis. For N9, no histological signs of neuroinflammation were noted. Captive foxes N3 and N6 were positive for vulpine circovirus (FoxCV) alone in brain tissue; pseudo-control foxes (where C denotes control foxes) C2 and C6 returned borderline results, defined as amplifying close to the assay cycle threshold cut-off with low efficiency and with two of the triplicate reactions returning signal (these samples were considered negative in all subsequent analyses). Of note, no FoxCV PCR-positive foxes tested positive for DogCV exposure by ELISA, indicating little to no cross-reactivity of the DogCV ELISA with a related circovirus species. Considering all captive foxes for which FoxCV tests had been performed, there was reasonably strong evidence of a higher FoxCV prevalence in captives versus matched pseudo-controls (66.7% (6/9) and 11.1% (1/9) respectively; *χ* ^2^ = 5.84, p = 0.038; **Table 3**).

**Table 3:** PCR and histology for foxes tested for various neurotropic pathogens using brain samples.

Fox ID	FoxCV*	*T. gondii*	*A. vasorum*	Histological inflammation
N1	−	−	−	N/A
N2	2.6 × 10^4^	+	−	+
N3	265	−	−	N/A
N4	2072	+	−	+
N5	−	−	−	N/A
N6	6.0 × 10^6^	−	−	+
N7	492	+	−	−
N8	25	−	+	+
N9	−	−	−	−
C1	237	+	+	+
C2	28^†^	−	−	−
C3	−	−	+	−
C4	−	−	−	−
C5	−	−	−	−
C6	13^†^	−	−	−
C7	−	−	−	−
C8	−	−	−	−
C9	−	−	−	−

Template copy numbers are listed for quantitative PCR reactions (FoxCV). Positive and negative symbols indicate results from end-point PCR reactions and assessment of histological inflammation. Fox IDs N1-9 indicate neurologically affected captive foxes and C1-9 indicate (presumed healthy) cull pseudo-control foxes. N/A, not available.

*Detection limit 10−100 template copies.

^†^Borderline results amplifying close to the cycle threshold with low efficiency and with two of the triplicate reactions returning signal. Considered as negative in analyses.

Having confirmed that DFS was associated with both *T. gondii* and FoxCV infection, we next looked to determine whether prevalence of co-infection differed between captive and pseudo-control fox populations, and thus whether DFS could result from neurotropic co-infection. Both the captive and pseudo-control populations showed a degree of co-infection: N2, N4, and N7 were positive for both FoxCV and *T. gondii*; and N8 and C1 were positive for both FoxCV and *A. vasorum* (C1 was also co-infected with *T. gondii*) (**Table 3**). There was a higher prevalence of co-infection with any agent in captive compared to pseudo-control foxes, with respective estimates of 44.4% (4/9; 3 FoxCV/*T. gondii*, 1 FoxCV/*A. vasorum*) and 11.1% (1/9; FoxCV/*T. gondii*/*A. vasorum*) (*χ* ^2^ = 2.49, p = 0.30). The overall prevalence of *T. gondii*/FoxCV co-infection in captive compared to pseudo-control foxes was 33.3% (3/9) vs. 11.1% (1/9) (*χ* ^2^ = 1.29, p = 0.57).

Sequencing of FoxCV PCR products from N2 and N3 confirmed specificity of the primers and amplification of the expected region of the genome. BLAST searches revealed 94% sequence identity with the three strains previously documented (48) (GenBank accession nos. KP260925–7) and 92% sequence identity with DogCV strains documented by Li and colleagues (61) (GenBank accession nos. KC241982–4).

### Gross Necropsy Examination and Histology

**Figure 1:**
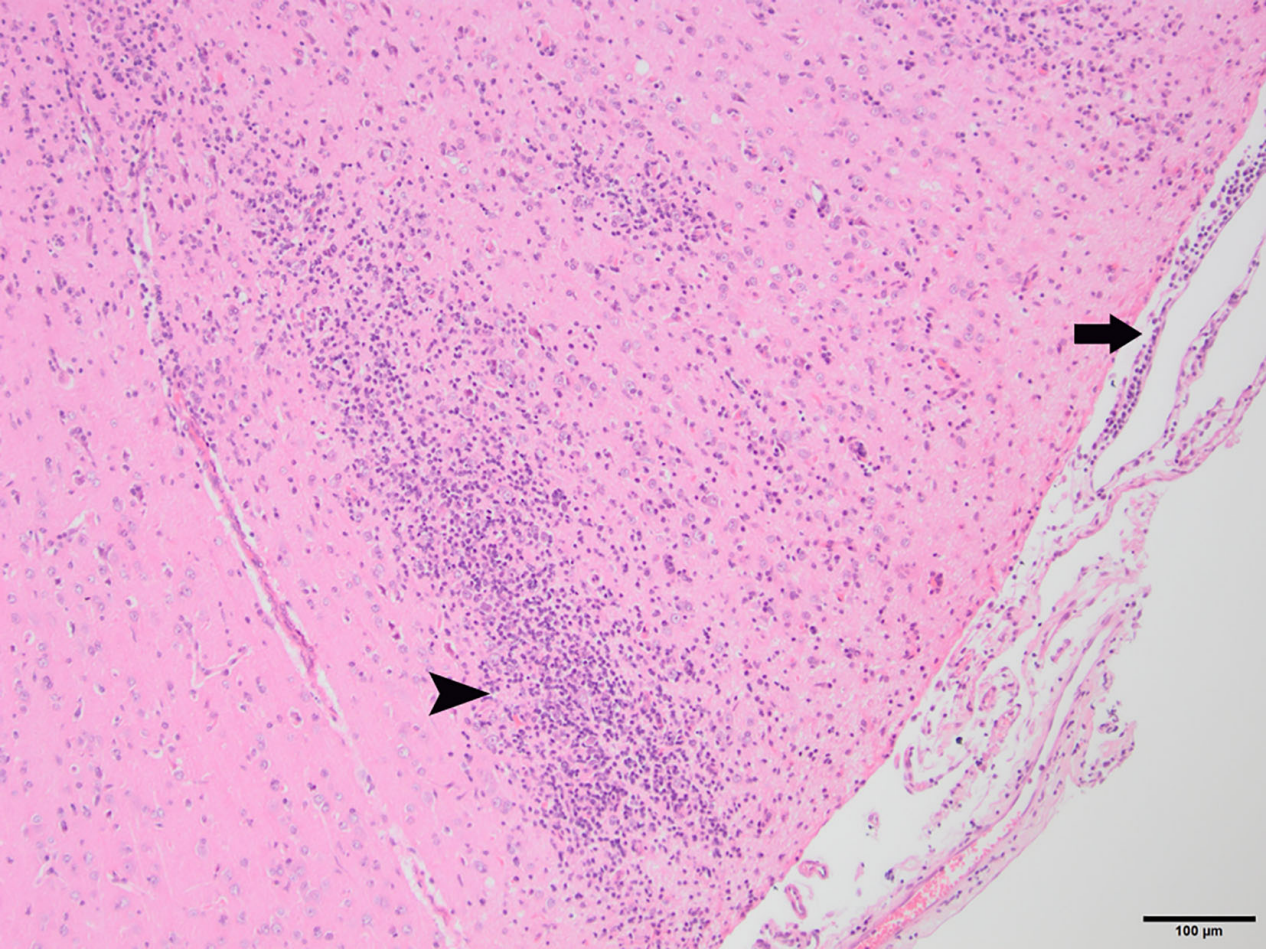
N2, H&E stain, 100×: Inflammation of the superficial grey matter with primarily lymphocytic infiltrates (non-suppurative polioencephalitis; arrowhead) with meningitis (arrow). Moderate gliosis and satellitosis are also present. At higher magnifications, shrunken, angular, hyper-eosinophilic neuronal cell bodies are observed, consistent with neuronal degeneration and early necrosis.

**Figure 2:**
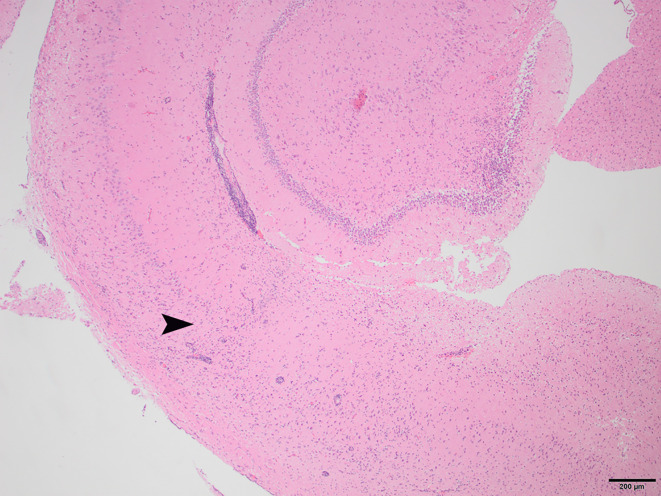
N4, H&E stain, 40×: Non-suppurative encephalitis at the border of the cortical white and gray matter. Inflammatory infiltrates are primarily lymphocytic, with a small proportion of plasma cells. The arrowhead indicates the border where the normal structure of the hippocampus becomes disrupted by infiltrating inflammatory cells.

**Figure 3:**
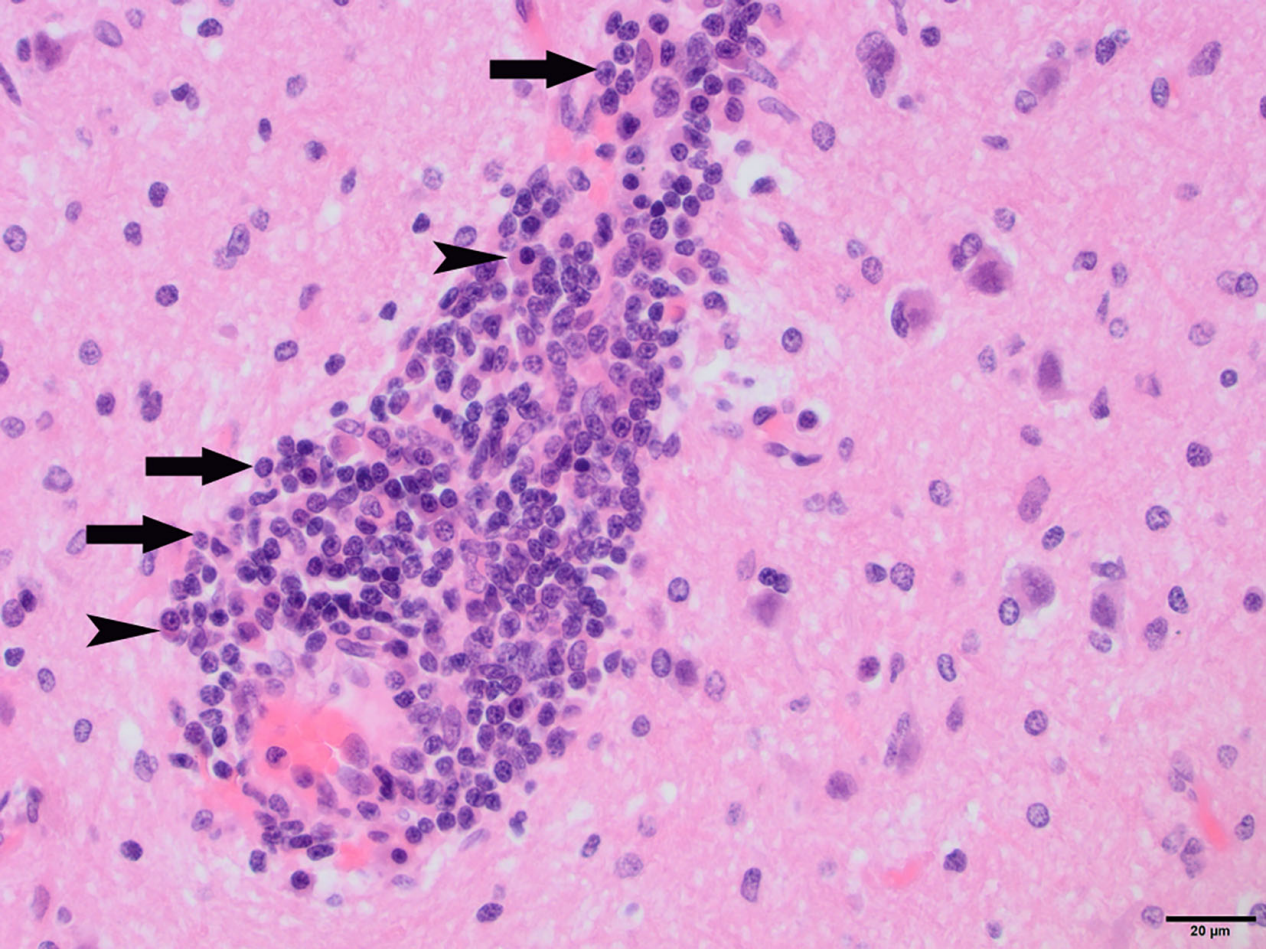
N4, H&E stain, 400×: Perivascular “cuff” composed primarily of lymphocytes (arrows) and plasma cells (arrowheads) filling the Virchow-Robins space.

**Figure 4:**
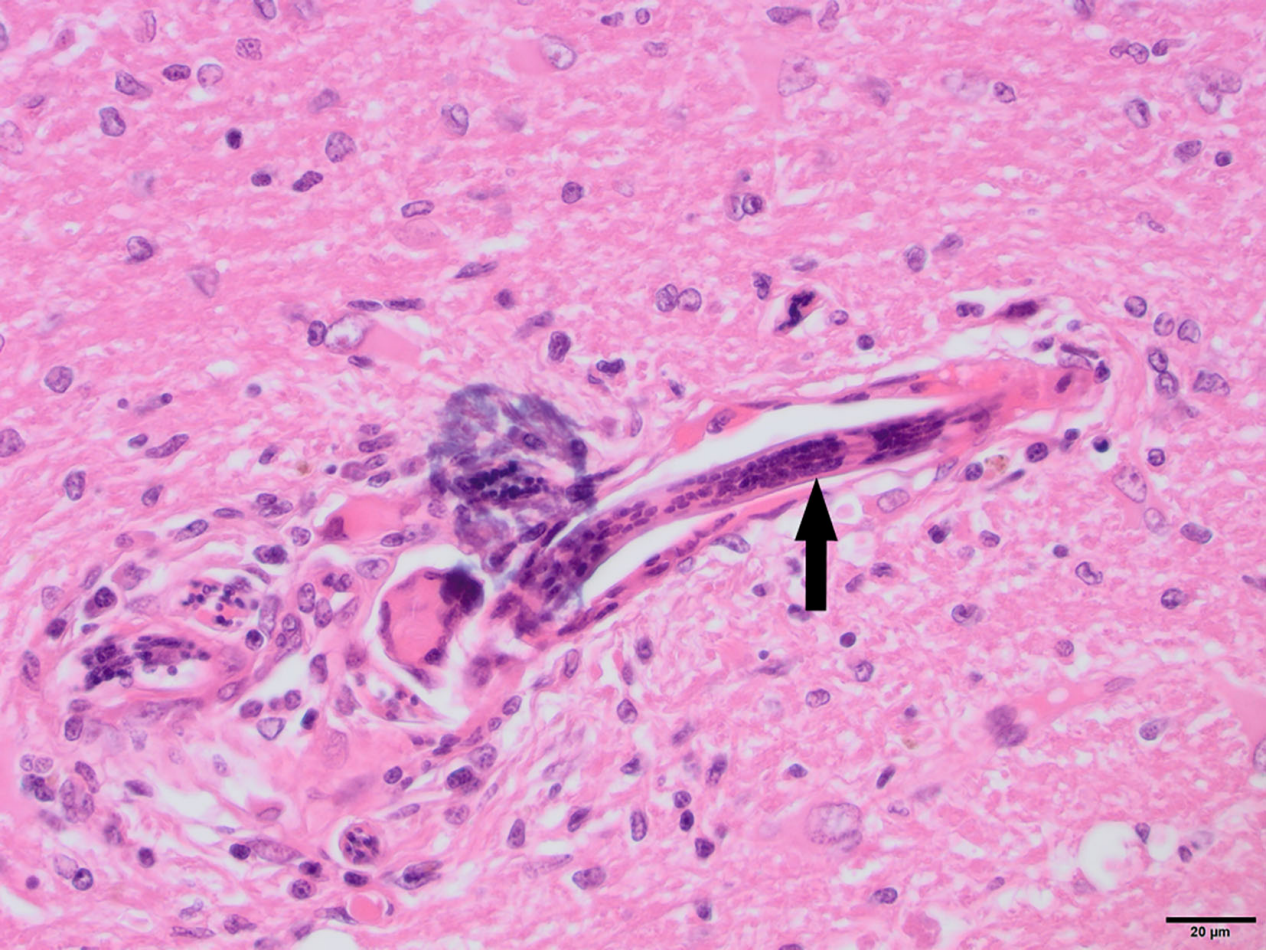
N8, H&E stain, 40×: Longitudinal section of *Angiostrongylus vasorum* larva within a blood vessel (arrow). Low numbers of lymphocytes, plasma cells and macrophages are present around the vessel. The presence of aberrant *A. vasorum* migration in the brain was confirmed with PCR. Multifocal hemorrhages and hemosiderin pigment were noted and suspected to be associated with aberrant larval migration.

**Figure 5:**
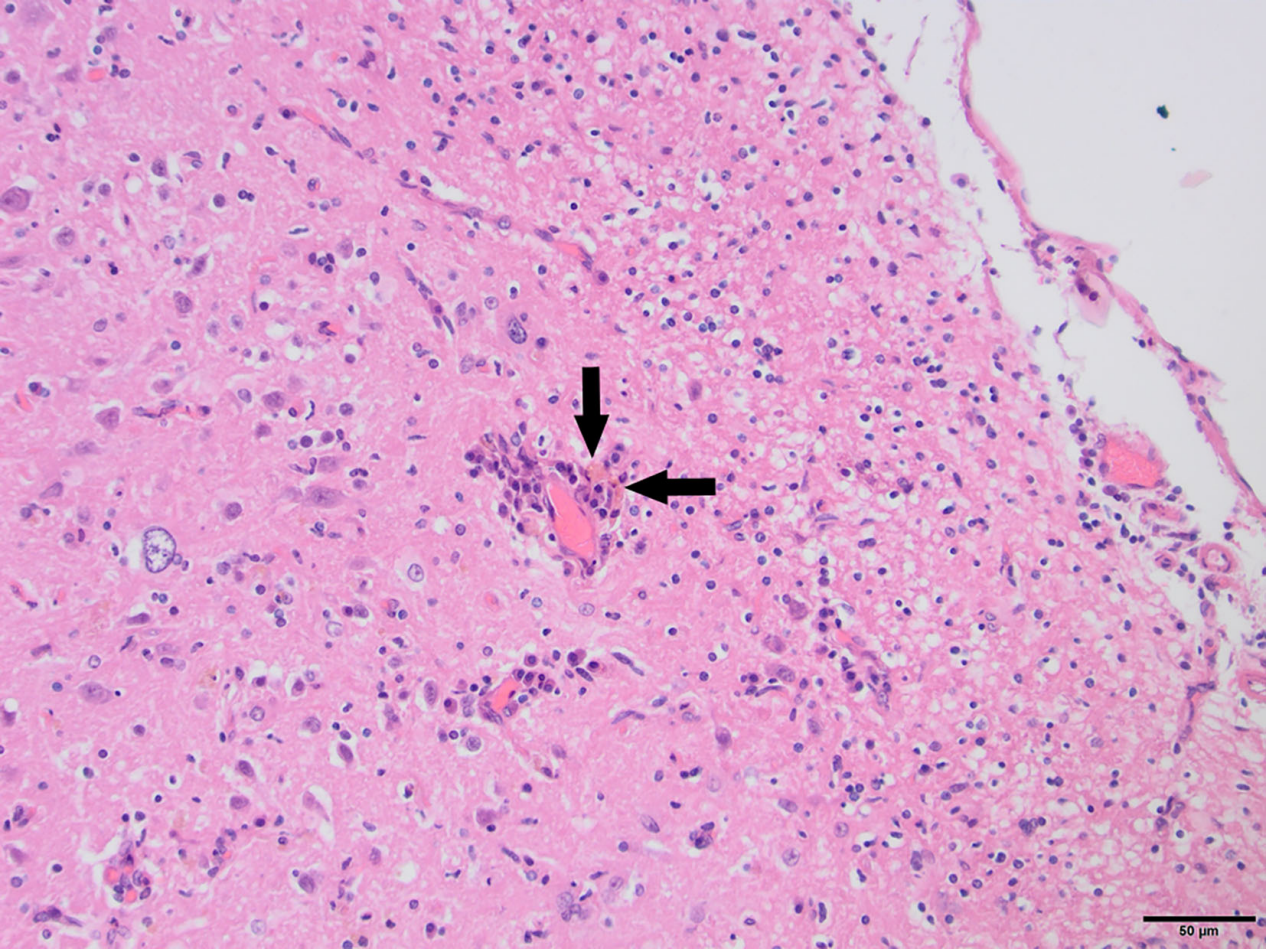
Fox C1, H&E stain, 200×: Mild perivascular cuffing and non-suppurative meningitis. Phagocytosed pigment (arrows) consistent with hemosiderin was observed in the cortical tissue as well as meninges, suggesting the presence of small multifocal hemorrhages.

To determine whether DFS was associated with neurological inflammation, gross necropsy was performed on a total of 83 foxes. For 15 foxes (six captive neurologically affected foxes and nine pseudo-control foxes), paired histology and infection status data were available for a range of neurotropic agents, excluding three captive foxes for which there were no formalin fixed samples (excluded from further analysis). Histological evidence of non-suppurative inflammation was detected in 5 samples: N2, N4, N6, and N8 showed lesions consistent with meningoencephalitis largely centered on the grey matter (polioencephalitis) (**Figures 1** **–** **4**), while C1 showed meningitis alone without concurrent involvement of the neuropil (**Figure 5**). All five affected tissues displayed prominent perivascular cuffing of inflammatory cells, with varying degrees of infiltration of the neuropil. Inflammatory infiltrates ranged from mainly lymphocytic (N2, **Figure 1**) to mixed lymphoplasmacytic (N4, **Figures 2**, **3**; N8, **Figure 4**). Changes included mild gliosis, mild satellitosis, and neuronal changes, including hypereosinophilia, shrinkage, and angular cell bodies indicative of degeneration. N4 in particular showed marked effacement of the normal hippocampal architecture (**Figure 2**). On histological examination of sections from N8 (**Figure 4**), the presence of an intravascular larva was noted, prompting the addition of the *A. vasorum* PCR assay.

Overall, histological inflammation was not evident for any of the six fox samples that were negative for all pathogens tested (5/9 pseudo-controls and 1/6 captives). Of the remaining nine foxes, eight were positive for FoxCV (five captives and three pseudo-controls). 80% (4/5) of FoxCV-infected captive foxes showed signs of histological inflammation (observed in in 2/3 cases of *T. gondii*/FoxCV co-infection, one singular FoxCV case, and one case of FoxCV/*A. vasorum* co-infection). 33.3% (1/3) of FoxCV-infected pseudo-controls showed signs of histological inflammation (FoxCV/*T. gondii*/*A. vasorum* co-infection). Singular FoxCV infection did not result in evident histological inflammation for C2 nor C6; but inflammation was observed for N6 which had particularly high FoxCV cerebral copy numbers (**Table 3**). There were no cases of singular infection with *T. gondii—*all 4 cases occurred in FoxCV-positive subjects. C3 was singularly infected with *A. vasorum* without evident histological inflammation. Therefore, 66.7% (4/6) of captive foxes showed signs of histological inflammation compared to 11.1% (1/9) of pseudo-control foxes (*χ* ^2^ = 5, p = 0.084).

### Fox Behavior in Relation to Infection Status

We next examined whether the fatal feline attraction behavior seen in *T. gondii*–infected rodent intermediate hosts is also found in *T. gondii*–infected foxes with DFS, using a modified version of a previously validated behavioral assay (22–24). Using a point-sampling approach in which six *T. gondii*–infected fox’s zonal location was recorded every 5 min, there was no evidence of a difference between the number of entries that foxes made into either the dog or cat zones before any odor was added versus after (Both dog and cat odor zones: *χ* ^2^ = 12, p = 1). However, after odor was added, there was suggestive evidence for an increased number of entries into the cat odor zones, relative to that within the dog odor zones (Friedman test: df = 20, p = 0.06).

### Biomarkers of Stress in Relation to Fox *T. gondii* Infection Status

**Figure 6:**
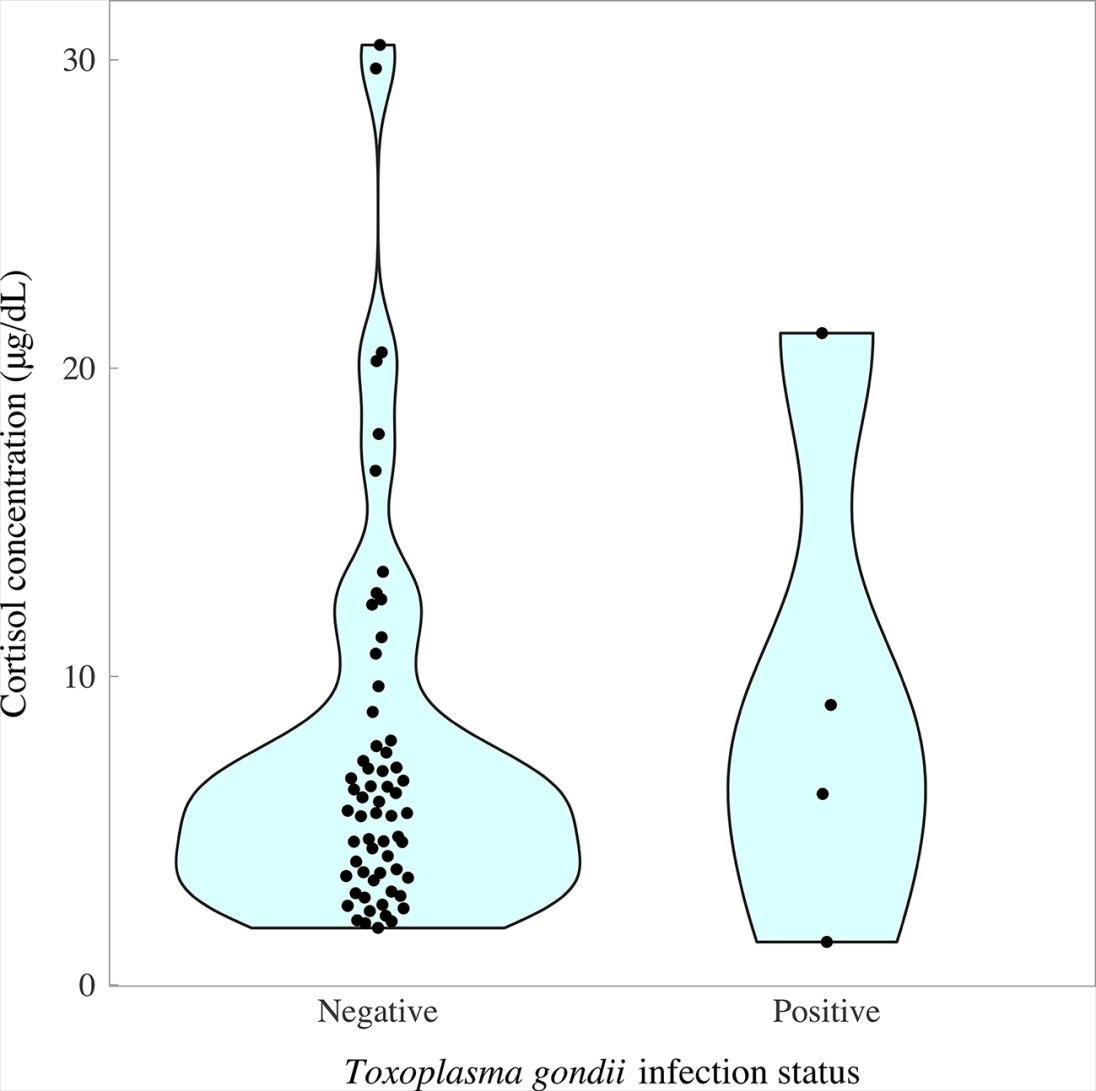
Concentration of cortisol in fox fur samples quantified by enzyme-linked immunosorbent assay in relation to *Toxoplasma gondii* infection status measured by nested polymerase chain reaction (*T. gondii* prevalence = 6.15%; 4/65). Colored areas represent the density distribution of the data.

**Figure 7:**
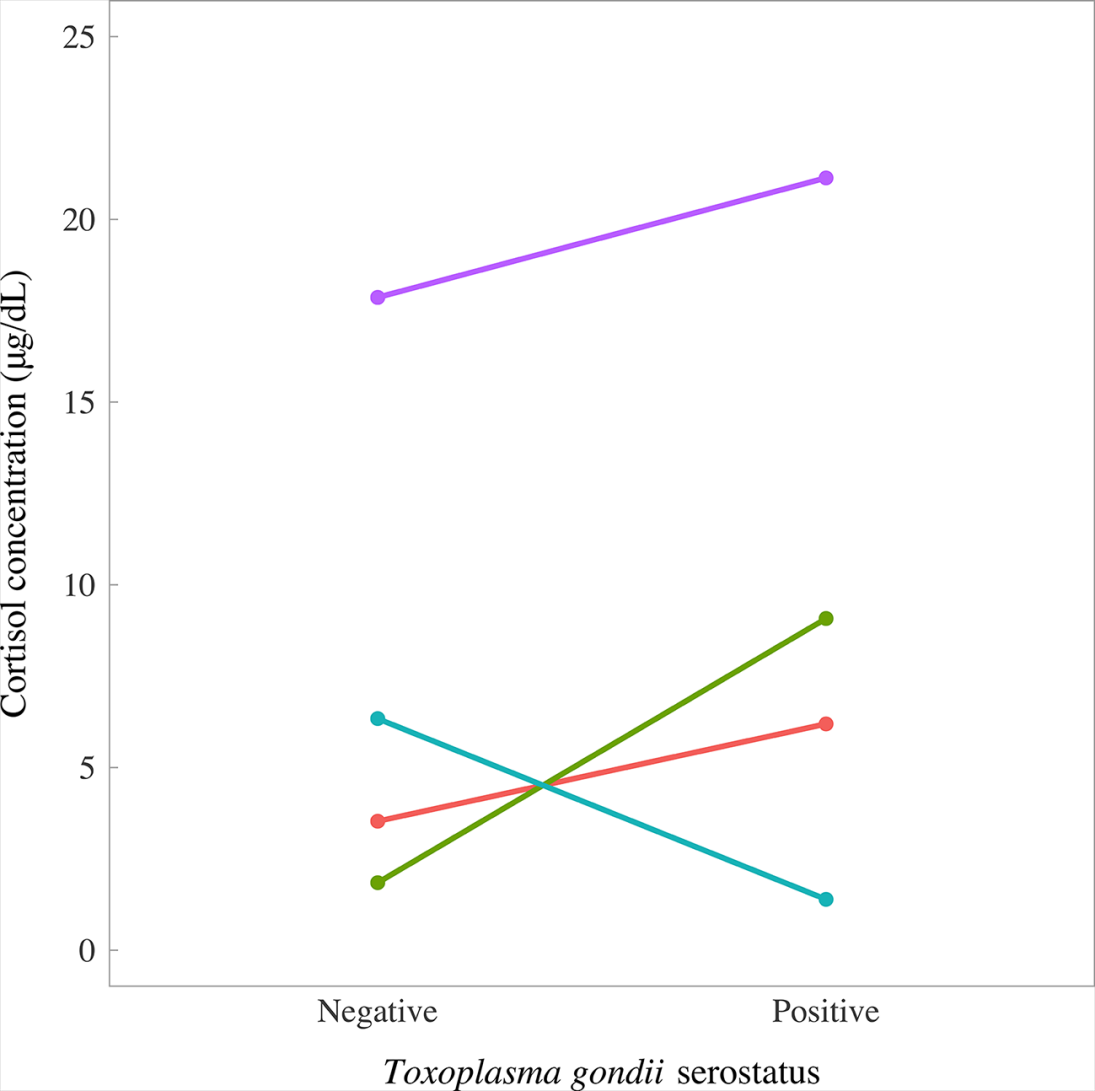
Concentrations of cortisol in fur samples of four matched pairs of foxes in relation to *Toxoplasma gondii* serostatus, measured by enzyme-linked immunosorbent assay. Foxes were matched for age, sex, and body condition score.

The cortisol content of fox fur was determined using a commercially available cortisol ELISA, as described previously for badger and dog (91, 92). Two samples were unavailable for *T. gondii* PCR testing because the brains were damaged during culling, leaving a total sample size of 65 pseudo-control foxes for which paired data were available on cortisol concentrations and *T. gondii* infection status. Cortisol concentrations followed a left-skewed distribution, with an interquartile range of 3.53–7.93 μg/dl and median of 7.93 μg/dl (overall range 1.39, 30.48 μg/dl). There was no evidence of a difference in cortisol concentrations between *T. gondii*-negative and *T. gondii-*positive foxes (*χ* ^2^ = 65, p = 0.89). However, there was a tendency for *T. gondii*-positive foxes to have slightly higher median concentrations of cortisol (*T. gondii*-negatives, 5.57 μg/dl (IQR 3.53, 7.75); *T. gondii*-positives, 7.64 μg/dl (IQR 4.99, 12.09); **Figure 6**). Paired foxes were matched for age, sex and body condition score to understand the effect of *T. gondii* infection on cortisol concentration in the absence of major confounders. This analysis revealed no evidence of a difference in cortisol concentration between matched pairs of *T. gondii* negative and positive foxes (Wilcoxon signed rank test: p = 0.63, n = 4 pairs), although there was a trend for slightly higher cortisol concentrations in *T. gondii* positive foxes compared to matched uninfected foxes in three out of four pairs (**Figure 7**).

## Discussion

In this series of trials, we aimed to test the hypotheses that the aberrant behavior of foxes with Dopey Fox Syndrome (DFS) is associated with infection with: a) *Toxoplasma gondii*; b) another infectious neurotropic agent; or c) *T. gondii* in addition to another infectious neurotropic agent (the two-hit hypothesis). Predictions were tested using a population of UK red foxes, including culled foxes (pseudo-controls) and live foxes housed in sanctuaries. We make the case that the red fox is a highly suitable model to examine naturally occurring parasite-altered host behaviors, especially those of no current evolutionary advantage (parasite constraint rather than parasite manipulation). More generally, this model system could help to elucidate possible links between chronic infection and neurological disorders across vertebrate hosts.

### Behavioral Abnormalities Associated With *T. gondii* Infection

We found support for the hypothesis that DFS is associated with *T. gondii* infection, since the prevalence of *T. gondii* was higher in captive compared to pseudo-control foxes, with respective estimates of 33.3% (3/9) and 6.8% (5/74). Captive foxes used in this study were brought into sanctuaries generally as a result of aberrant behaviors, spanning from reduced fear of humans to more pathological signs including anorexia and circling behaviors, which are all consistent with DFS. In contrast, pseudo-control foxes were those shot by pest control companies and thus could be argued to be a reasonably representative sample of the UK healthy wild fox population. We do acknowledge, however, that 6.8% of our pseudo-control (non-captive) foxes shot by pest-controllers were *T. gondii* positive, which is below the documented 20% average for the UK fox population (93). This lower than expected prevalence could be due to the high number of young foxes included in this study: where age data were available, 37% (24/65) were under a year old and so their likelihood of encountering *T. gondii*—either by consumption of infected meat or *via* contact with oocysts in the environment—would be lowered.

Further support for the association of *T. gondii* with DFS was provided by our behavioral analyses which indicated a preference of foxes for cat odor zones compared to dog odor zones among infected individuals. While sample size was small (six foxes), this finding tentatively supports an expansion of the fatal feline attraction (19) behavior as observed in rodents and other intermediate/secondary hosts (21, 22, 44). Qualitative observations also confirmed a lack of fear of humans and increased inquisitiveness among *T. gondii*-seropositive captive foxes with DFS. Further research using larger samples of foxes should be performed using a similar experimental design to confirm the biological significance of the current findings. Contrary to our prediction that *T. gondii*–infected foxes would display lower levels of cortisol (an indicator of chronic stress) compared to uninfected foxes, we found no significant evidence of an association between cortisol concentration and infection status in a sample of 65 red foxes. Nevertheless, in pairs of foxes matched for age, sex and body condition, there was a tendency for higher cortisol concentrations in *T. gondii*–infected animals in 3/4 pairs, relative to their uninfected counterparts, warranting further research in this area.

### Behavioral Abnormalities Associated With Other Neurotropic Infections

Consistent with a hypothesis of DFS being associated with another neurotropic infection, we also observed a higher prevalence of fox-specific circovirus (FoxCV) infection in captive compared to in pseudo-control foxes, with respective estimates of 66.7% (6/9) and 11.1% (1/9). However, with the exception of *A. vasorum*, we found no evidence of infection for any other tested neurotropic agents (canine distemper virus (CDV), canine adenovirus type-1 (CAV-1) and CAV-2). While these analyses are based on limited sample sizes, we argue that such consistent observations are likely to be based on biologically meaningful phenomena. Our study has thereby also both confirmed the presence of FoxCV in wild red foxes in the UK (59) and indicated a pattern of infection for further characterization. A circovirus was recently shown to be associated with disease in domestic dogs (76), with animals presenting primarily for signs of fibrinonecrotic vasculitis and hemorrhagic diarrhea. Similarly, DogCV has been isolated in relation to hemorrhagic gastroenteritis in domestic canids of varying geographic origin (94–96). Gross lesions including necrotizing lymphadenitis and vasculitis have also been described, similar to porcine dermatitis and nephropathy syndrome (97). Bexton and colleagues (59) demonstrated that approximately 50% of behaviorally affected foxes were positive for FoxCV alone using PCR and immunohistochemistry; although FoxCV was also detected in serum from a proportion of neurologically normal foxes. This could also help to explain our finding of a single pseudo-control fox positive for FoxCV, with a high cerebral copy number and neurological inflammation.

### Behavioral Abnormalities Associated With *T. gondii* and Co-Infecting Neurotropic Agent(s)

Perhaps indicative of a synergistic role of *T. gondii* in neurological disease, in our study *T. gondii* infections always occurred in concert with another neurotropic agent. Most notably, we observed a higher prevalence of *T. gondii*/FoxCV co-infections in captive foxes (33.3%, 3/9) compared to pseudo-control foxes (11.1%, 1/9); although sample sizes provided little statistical power to definitively discriminate between groups. The prevalence of co-infection with any pathogen was also higher in the captive population (44.4%, 4/9) compared to pseudo-controls (11.1%, 1/9). These findings tentatively support our hypothesis that the aberrant behaviors of foxes with DFS may result from co-infection of *T. gondii* with another neurotropic agent—in particular that of FoxCV. Circoviruses are known to be immunosuppressive pathogens (69, 98, 99), so one could expect infection with FoxCV to not only increase host susceptibility to other infections, including *T. gondii*, but also to increase the probability of reactivation of latent *T. gondii* infection. Indeed, perhaps indicative of an immunosuppressive role of FoxCV, our serological data showed repeated anti-*T. gondii* IgG seroreversions and seroconversions among several captive foxes. In keeping with an immunosuppressive/co-infecting role of circoviruses, surveys across a number of domestic canines and wild carnivores in Italy showed that all DogCV-infected animals were co-infected with at least one other agent (68). Potentially in keeping with this hypothesis, in our study, histological inflammation was noted 80% (4/5) of FoxCV co-infections.

Our findings thus suggest that FoxCV infection may act synergistically with *T. gondii* to cause neuroinflammatory conditions in hosts with or without additional co-infecting neurotropic agent(s). In terms of behavioral and neurological disorders, these findings suggest that *T. gondii* infection may play a secondary role in their development (infection upon host immunosuppression), but that *T. gondii* infection may increase the severity of inflammation and thus perhaps the severity of clinical disease. Though statistical power was limited due to small samples, these preliminary findings tentatively support our hypothesis for a two (or more)-hit model of neurological impairment. Subject to further corroboration, these results suggest that *T. gondii* and FoxCV co-infection may play an important role in the development of DFS in *V. vulpes*.

Within this multiple-hit model framework, it may also be important to consider timing of infection. For example, in humans, it is known that the likelihood of *T. gondii* congenital transmission increases with increasing maternal gestational age at infection. Moreover, increasing evidence suggests that maternal *T. gondii* infection during the perinatal period and the subsequent inflammatory response may be, in part, responsible for the onset of severe neuropsychiatric disorders, including schizophrenia, in the offspring’s adolescence or early adulthood (100, 101). Further work should therefore look to identify whether timing of infection is indeed important in influencing the presence or outcome of neurological disease. In addition, it may be valuable to examine whether the infectious dose influences the onset or maintenance of disease and whether there are specific time-lags between fox (co-)infection and neurological disease onset. These additional insights could be gained by collecting population-level longitudinal data on the seroprevalence of various infectious diseases as well as clinical symptoms of foxes. Identifying a consistency and temporality of exposure–outcome association will strengthen the hypothesis presented here.

## Concluding Remarks

Here, we highlight the potential of using the red fox, *Vulpes vulpes*, as a model system to shed light on infectious agent factors underlying the occurrence of behavioral and neurological abnormalities in vertebrate hosts. As evidenced by our infection prevalence and histological findings, comparing presumed neurologically normal foxes to foxes with symptoms consistent with Dopey Fox Syndrome, the current study puts evidence toward a two-hit model of neurological disease development in which host infection with neurotropic agents, including *T. gondii* and circovirus and possibly other neurotropic infections, may cause host predisposition to neurological disease. Overall, while pilot in nature, the current study brings forth new questions regarding the extent to which specific neurotropic agents influence neurologic disease onset and maintenance, and how infection may alter host physiology and behavior in general.

## Data Availability Statement

All datasets generated for this study are included in the article/supplementary material.

## Ethics Statement

The animal study was reviewed and approved by the Royal Veterinary College Clinical Research and Ethical Review Board (CREB) (URN numbers: 2018 RP2_004-2 and 2017 RP2_1768-3).

## Author Contributions

JW, SP, CF, TB, and HP conceived the study designs. CF, TB, HP, CT, EL, CMH, JW, RCF, and MH collected the data. GM, JW, CF, TB, and HP contributed to the data analysis and interpretation. GM and JW wrote the first draft. All authors contributed to the article and approved the submitted version.

## Funding

We acknowledge funding from the Royal Veterinary College and the Biotechnology and Biological Sciences Research Council (BBSRC) (London Interdisciplinary Doctoral Training Programme funding to GM [grant number BB/M009513/1], under the supervision of MW and JW), together with a London International Development Centre (LIDC) pump-priming grant (to MW and JW).

## Conflict of Interest

The authors declare that the research was conducted in the absence of any commercial or financial relationships that could be construed as a potential conflict of interest.
